# Supplementary material for: Content-rich biological network constructed by mining PubMed abstracts
Source: BMC Bioinformatics. 2004 Oct 8;5:147. doi: 10.1186/1471-2105-5-147 (PMC528731; doi:10.1186/1471-2105-5-147)
Supplement: Additional File 2 — The original results of the above study (non-essential files are deleted to keep the file size under the limit set by BMC bioinformatics). [file 1471-2105-5-147-S2.bz2 › chilibotAdditionalFile2/dip10/370ID9045692E6045/html/FGF7_FGFR3.html]

 


 **FGF7** and **FGFR3** 
  
Found 22 abstracts in PubMed, retrieved 10.  
 

 What does Google say? 
 PDF only 
| .edu only 

---

- Cancer Lett, 2003   **Loss of ligand binding specificity of fibroblast growth factor receptor 2 by RNA splicing in human chondrosarcoma cells.** .
  We have previously reported the alternatively spliced transcripts of fibroblast growth factor receptor FGFR 3 **[FGFR3]** derived by aberrant splicing in human cancers.
  Here, we describe a novel splice variant of FGFR2 FGFR2DeltaIII arising from skipping exons 7 10, resulting in the deletion of Ig like III domain in human chondrosarcoma cell.
  Sf9 cells expressing FGFR2DeltaIII were able to bind FGF1, FGF2, and **FGF7**, leading to loss of ligand binding specificity.
  Together with our previous findings, the present studies suggest that mRNA splicing plays an important role in the regulation of FGFRs function.

  - Cleft Palate Craniofac J, 2002   **Toward pathogenesis of Apert cleft palate FGF, FGFR, and TGF beta genes are differentially expressed in sequential stages of human palatal shelf fusion.** .
    OBJECTIVE Critical cellular events at the palatal medial edge epithelium MEE occur in unperturbed mammalian palatogenesis, the molecular control of which involves a number of growth factors including transforming growth factor beta 3 TGF beta 3 .
    Apert syndrome is a monogenic human disorder in which cleft palate has been significantly correlated to the fibroblast growth factor receptor FGFR 2 Ser252Trp mutation.
    We report the relative expression of these genes in human palatogenesis.
    METHODS The expression of the IgIIIa b and IgIIIa c transcript isoforms of FGFR2 and the proteins FGFR1, FGFR2, and **FGFR3** was studied in situ throughout the temporospatial sequence of human palatal shelf fusion and correlated with the expression of TGF beta 3.
    In addition, the immunolocalization of the ligand FGFs , and 7 was undertaken together with the intracellular transcription factor STAT1, which is activated by FGFR signaling.
    RESULTS FGFRs are differentially expressed in the mesenchyme and epithelia of fusing palatal shelves, in domains overlapping those of their ligands FGF4 and FGF2 but not **FGF7**.
    Coexpression is seen with TGF beta 3, which is implicated in MEE dynamics and FGF and FGFR upregulation, and STAT1, an intracellular transcription factor that mediates apoptosis.
    CONCLUSIONS The coregulation of molecules of the FGFR signaling pathway with TGF beta 3 throughout the stages of human palatal fusion suggests their controlling influence on apoptosis and epitheliomesenchymal transdifferentiation at the MEE.
    Experimental evidence links FGFR2 IgIIIa b loss of function with palatal clefting, and these correlated data suggest a unique pathological mechanism for Apert cleft palate.

    - Plast Reconstr Surg, 2001   **From genotype to phenotype the differential expression of FGF, FGFR, and TGFbeta genes characterizes human cranioskeletal development and reflects clinical presentation in FGFR syndromes.** .
      Mutations in the fibroblast growth factor receptor FGFR genes , and 3 are causal in a number of craniofacial dysostosis syndromes featuring craniosynostosis with basicranial and midfacial deformity.
      Great clinical variability is displayed in the pathologic phenotypes encountered.
      To investigate the influence of developmental genetics on clinical diversity in these syndromes, the expression of several genes implicated in their pathology was studied at sequential stages of normal human embryo fetal cranial base and facial ossification n = 6 .
      At 8 weeks of gestation, FGFR1, FGFR2, and **FGFR3** are equally expressed throughout the predifferentiated mesenchyme of the cranium, the endochondral skull base, and midfacial mesenchyme.
      Both clinically significant isoforms of FGFR2, IgIIIa c and IgIIIa b, are coexpressed in maxillary and basicranial ossification.
      By 10 to 13 weeks, FGFR1 and FGFR2 are broadly expressed in epithelia, osteogenic, and chondrogenic cell lineages.
      **FGFR3** is maximally expressed in dental epithelia and proliferating chondrocytes of the skull base, but poorly expressed in the osteogenic tissues of the midface.
      FGF2 and FGF4, but not **FGF7**, and TGFbeta1 and TGFbeta3 are expressed throughout both osteogenic and chondrogenic tissues in early human craniofacial skeletogenesis.
      Maximal FGFR expression in the skull base proposes a pivotal role for syndromic growth dysplasia at this site.
      Paucity of **FGFR3** expression in human midfacial development correlates with the relatively benign human mutant **FGFR3** midfacial phenotypes.
      The regulation of FGFR expression in human craniofacial skeletogenesis against background excess ligand and selected cofactors may therefore play a profound role in the pathologic craniofacial development of children bearing FGFR mutations.

      - J Neurosurg, 2001   **Negative autoregulation of fibroblast growth factor receptor 2 expression characterizing cranial development in cases of Apert P253R mutation and Pfeiffer C278F mutation syndromes and suggesting a basis for differences in their cranial phenotypes.** .
        OBJECT Heterogeneous mutations in the fibroblast growth factor receptor 2 gene FGFR2 cause a range of craniosynostosis syndromes.
        The specificity of the Apert syndrome affected cranial phenotype reflects its narrow mutational range 98% of cases of Apert syndrome result from an Ser252Trp or Pro253Arg mutation in the immunoglobulin like Ig IIIa extracellular subdomain of FGFR2.
        In contrast, a broad range of mutations throughout the extracellular domain of FGFR2 causes the overlapping cranial phenotypes of Pfeiffer and Crouzon syndromes and related craniofacial dysostoses.
        METHODS In this paper the expression of FGFR1, the IgIIIa c and IgIIIa b isoforms of FGFR2, and **FGFR3** is investigated in Apert syndrome P253R mutation and Pfeiffer syndrome C278F mutation affected fetal cranial tissue and is contrasted with healthy human control tissues.
        Both FGFR1 and **FGFR3** are normally expressed in the differentiated osteoblasts of the periosteum and osteoid, in domains overlapped by that of FGFR2, which widely include preosseous cranial mesenchyme.
        Expression of FGFR2 is restricted to domains of advanced osseous differentiation in both Apert syndrome and Pfeiffer syndrome affected cranial skeletogenesis in the presence of fibroblast growth factor FGF 2, but not in the presence of FGF4 or **FGF7**.
        Whereas expression of the FGFR2 IgIIIa b KGFR isoform is restricted in normal human cranial osteogenesis, there is preliminary evidence that KGFR is ectopically expressed in Pfeiffer syndrome affected cranial osteogenesis.
        CONCLUSIONS Contraction of the FGFR2 IgIIIa c BEK expression domain in cases of Apert syndrome and Pfeiffer syndrome affected fetal cranial ossification suggests that the mutant activation of this receptor, by ligand dependent or ligand independent means, results in negative autoregulation.
        This phenomenon, resulting from different mechanisms in the two syndromes, offers a model by which to explain differences in their cranial phenotypes.

        - Kidney Int, 2001   BACKGROUND Fibroblast growth factors are a family of at least 21 heparin binding proteins involved in many biological processes, both during development and in the adult, including cell proliferation, differentiation, and angiogenesis.
          FGFs mediate their effects through high affinity tyrosine kinase receptors , which are encoded by four genes.
          The aims of the present study were to localize FGFR 1 through FGFR 3 **[FGFR3]** in the normal adult rat kidney and to determine which functional FGFR variants and FGFs were expressed.
          METHODS Avidin biotin enhanced horseradish peroxidase immunohistochemistry was used on paraffin sections of rat kidney to localize FGFR 1 through FGFR 3 **[FGFR3]**.
          . . . reverse transcriptase polymerase chain reaction was used to examine expression of the receptor variants and also of FGF 1 through FGF 10 in cortex, outer medulla, and inner medulla.
          RESULTS By immunohistochemistry, each receptor was localized to distinct and overlapping nephron segments, such that one or more FGFRs were localized to all nephron and collecting duct epithelia.
          FGFR 1 and FGFR 3 **[FGFR3]** were localized to glomeruli, FGFR 3 **[FGFR3]** to proximal tubules and FGFR 1 to thin limbs.
          FGFR 1 through FGFR 3 **[FGFR3]** were localized to distal straight tubules, with FGFR 1 and FGFR 3 **[FGFR3]** localized to distal convoluted tubules.
          FGFR 1 and FGFR 3 **[FGFR3]** were localized to medullary collecting ducts.
          In addition, FGFR 1 was localized to the smooth muscle of renal arteries.
          All seven FGFR variants were expressed in the cortex and outer medulla, with fewer FGFRs in the inner medulla.
          FGF 1, FGF 2, FGF 7 **[FGF7]**, FGF 8, and FGF 9 were expressed in the kidney, with FGF 10 expression found only in the cortex.
          CONCLUSIONS Mapping of these receptors is critical to the determination of the effects of FGF ligands in discrete regions of the kidney.
          The distributions of the FGFRs in the normal adult kidney and the restricted expression of FGF ligands suggest that specific FGFs have distinct and important roles in the maintenance of normal kidney structure and function.

          - Hear Res, 2001   **The expression of fibroblast growth factors and their receptors in the embryonic and neonatal mouse inner ear.** .
            Four different fibroblast growth factor receptors FGFR are known, three of which have splice variants known as the b and c variants in the FGF binding domain, to give different patterns of sensitivity to the different FGFs.
            The expression of the b and c variants of the FGF receptors, together with the expression of the ligands FGF1, FGF2, FGF3, **FGF7**, FGF8b and FGF8c, was determined by quantitative reverse transcription polymerase chain reaction in developing whole mouse inner ears, and in dissected components of the postnatal mouse inner ear.
            At embryonic age E 10.5 days, when the otocyst is a simple closed sac, the receptor most heavily expressed was FGFR2b, relative to the postnatal day 0 level.
            Over the period E10.5 E12.5, during which the structures of the inner ear start to form, the expression of the different FGF receptors increased 10 2 10 4 fold per unit of tissue, and there was a gradual switch towards expression of the c splice variants of FGFR2 and **FGFR3** rather than the b variants.
            At E10.5, the ligands most heavily expressed, relative to the postnatal day 0 level, were FGF3, FGF8b and FGF8c.
            In the postnatal inner ear, the patterns of expression of receptors and ligands tended to be correlated, such that receptor variants were expressed in the same regions as the ligands that are known to activate them effectively.
            The neural sensory region expressed high levels of FGFR3c, and high levels of the ligand FGF8b.
            The same area also expressed high levels of FGFR1b and FGFR2b, and high levels of FGF3.
            The lateral wall of the cochlea including the stria vascularis and the spiral ligament expressed high levels of FGFR1c and FGF2.
            It is suggested that the different FGF receptors and ligands are expressed in a spatially coordinated pattern, to selectively program cochlear development.

            - In Vitro Cell Dev Biol Anim, 2000   **Isolation and serum free culture of epithelial cells derived from epithelial rests of Malassez in human periodontal ligament.** .
              To study biological character and function of epithelial rests of Malassez ERM in human periodontal ligament, we have developed a serum free culture system of epithelial cells ME derived from ERM.
              The mitogenic effects of fibroblast growth factor FGF 1, FGF 2, and FGF 7 **[FGF7]** keratinocyte growth factor **[FGF7]** KGF **[FGF7]** on ME, human periodontal ligament derived fibroblasts PLF , human oral epithelial cells OE , and human submandibular gland derived epithelial cells SGE were investigated under a serum free culture condition.
              FGF 1 and FGF 7 **[FGF7]** KGF **[FGF7]** stimulated the growth of both ME and SGE but FGF 2 had no effect.
              On the other hand, FGF 1, FGF 2, and FGF 7 **[FGF7]** KGF **[FGF7]** increased the OE proliferation.
              These results suggested that the divergent requirement of FGF ligands among these cells would be attributed to the different expression pattern of FGF receptor FGFR messenger ribonucleic acid mRNA isotypes.
              Therefore, we examined the expression of FGFR isotypes in these cells.
              Reverse transcription polymerase chain reaction RT PCR analysis of ME and SGE derived mRNAs revealed the presence of mRNA encoding FGFR2 IIIb, a high affinity receptor for FGF 1 and FGF 7 **[FGF7]** KGF **[FGF7]**.
              However, no mRNAs for other FGFR isotypes were detected in both ME and SGE.
              On the contrary, OE expressed FGFR1 IIIc, **FGFR3** IIIb, and FGFR4 mRNAs as well as FGFR2 IIb.
              These results indicate that FGF binding sites on ME dominantly bind to FGF 1 and FGF 7 **[FGF7]** KGF **[FGF7]**, which transduce their signals via FGFR2 IIIb.
              Immunohistochemical analysis, PCR Southern, ribonuclease protection assay RPA , and Western blotting revealed that PLF expressed FGF 7 **[FGF7]** KGF **[FGF7]** mRNA and its peptide.
              These observations suggest that FGF 7 **[FGF7]** KGF **[FGF7]** might mediate epithelial mesenchymal interactions between ME and PLF to maintain normal structure and function of periodontal ligament.

              - J Cancer Res Clin Oncol, 2000   **Up regulation and co expression of fibroblast growth factor receptors in human gastric cancer.** .
                Fibroblast growth factor FGF , a key regulatory factor of cell growth and differentiation, is involved in embryonic development, angiogenesis, and tumorigenesis.
                To date, four different FGF receptors FGFRs have been cloned and characterized.
                We examined the expression of four FGFRs in human gastric cancer tissues and cell lines using Northern analysis, ribonuclease protection assay, and immunohistochemistry.
                The mRNAs of FGFR 1 10 14 , FGFR 2 9 14 , and FGFR 4 9 14 were up regulated in cancer compared with normal tissues.
                FGFR 3 **[FGFR3]** mRNAs were barely detectable in both normal and cancer tissues.
                These FGFR mRNAs were co expressed in various combinations of two or three in the same tissue.
                Immunohistochemistry confirmed specific staining of multiple FGFRs, except FGFR 3 **[FGFR3]**, in the cancer specimens.
                To investigate the functional significance of FGFR co expression we examined the invasive property of SNU 16 cells, which exhibited gene amplification of FGFR , and 4 as well as over expression of keratinocyte growth factor **[FGF7]** receptor KGFR , a splice variant of FGFR 2, and FGFR 4 mRNA.
                KGF **[FGF7]** plus acidic FGF aFGF , KGF **[FGF7]**, and aFGF treatment enhanced the invasive potential of SNU 16 cells over the control by 100%, 107%, and 47%, respectively, indicating that neither additive nor synergistic effect was induced by stimulation with aFGF plus KGF **[FGF7]**.
                These results suggest that co expression of FGFRs in various combinations may cause subtle changes in the progression of gastric cancer.

                - J Histochem Cytochem, 2000   **Gene expression patterns of the fibroblast growth factors and their receptors during myogenesis of rat satellite cells.** .
                  Satellite cells are the myogenic precursors in postnatal muscle and are situated beneath the myofiber basement membrane.
                  We previously showed that fibroblast growth factor 2 FGF2, basic FGF stimulates a greater number of satellite cells to enter the cell cycle but does not modify the overall schedule of a short proliferative phase and a rapid transition to the differentiated state as the satellite cells undergo myogenesis in isolated myofibers.
                  In this study we investigated whether other members of the FGF family can maintain the proliferative state of the satellite cells in rat myofiber cultures.
                  We show that FGF1, FGF4, and FGF6 as well as hepatocyte growth factor, HGF enhance satellite cell proliferation to a similar degree as that seen with FGF2.
                  . . . FGF5 and **FGF7** are ineffective.
                  None of the growth factors prolongs the proliferative phase or delays the transition of the satellite cells to the differentiating, myogenin state.
                  However, FGF6 retards the rapid exit of the cells from the myogenin state that routinely occurs in myofiber cultures.
                  To determine which of the above growth factors might be involved in regulating satellite cells in vivo, we examined their mRNA expression patterns in cultured rat myofibers using RT PCR.
                  The expression of all growth factors, excluding FGF4, was confirmed.
                  Only FGF6 was expressed at a higher level in the isolated myofibers and not in the connective tissue cells surrounding the myofibers or in satellite cells dissociated away from the muscle.
                  By Western blot analysis, we also demonstrated the presence of FGF6 protein in the skeletal musle tissue.
                  Our studies therefore suggest that the myofibers serve as the main source for the muscle FGF6 in vivo.
                  We also used RT PCR to analyze the expression patterns of the four tyrosine kinase FGF receptors FGFR1 FGFR4 and of the HGF receptor c met in the myofiber cultures.
                  Depending on the time in culture, expression of all receptors was detected, with FGFR2 and **FGFR3** expressed only at a low level.
                  Only FGFR4 was expressed at a higher level in the myofibers but not the connective tissue cell cultures.
                  FGFR4 was also expressed at a higher level in satellite cells compared to the nonmyogenic cells when the two cell populations were released from the muscle tissue and fractionated by Percoll density centrifugation.
                  The unique localization patterns of FGF6 and FGFR4 may reflect specific roles for these members of the FGF signaling complex during myogenesis in adult skeletal muscle.

                  - J Biol Chem, 2000   Fibroblast growth factors transmit their signals through four transmembrane receptors that are designated FGFR1 4.
                    Alternative splicing in the extracellular region of FGFR1 3 generates receptor variants with different ligand binding affinities.
                    Thus two types of transmembrane receptors have been identified for FGFR2 and **FGFR3**, and the existence of analogous variants has been postulated for FGFR1 based on its genomic structure.
                    However, only a single full length transmembrane FGFR1 variant has been identified so far.
                    Here we describe the cloning of a full length cDNA encoding FGFR1 IIIb from a mouse skin wound cDNA library.
                    This receptor isoform was expressed at the highest levels in a subset of sebaceous glands of the skin and in neurons of the hippocampus and the cerebellum.
                    FGFR1 IIIb was expressed in L6 rat skeletal muscle myoblasts and used in cross linking and receptor binding studies.
                    FGF 1 was found to bind the receptor with high affinity.
                    . . . FGF 0, and 7 bound with significantly lower affinities.
                    Despite their apparently similar but low affinities, FGF 10 but not FGF 7 **[FGF7]** induced the activation of p44 42 mitogen activated protein kinase in FGFR1 IIIb expressing L6 myoblasts and stimulated mitogenesis in these cells, demonstrating that this new receptor variant is a functional transmembrane receptor for FGF 10.
